# Atypical Presentation of Systemic Amyloid Light Chain (AL) Amyloidosis

**DOI:** 10.7759/cureus.85777

**Published:** 2025-06-11

**Authors:** James Faraci, Joseph A Connor, Sukhbir Randhawa

**Affiliations:** 1 Department of Graduate Medical Education, Samaritan Medical Center, Watertown, USA; 2 Surgery, Lake Erie College of Osteopathic Medicine, Elmira, USA

**Keywords:** amyloidosis al, cardiac amyloidosis, cardiac amyloidosis with reduced ejection fraction, daratumumab, delayed diagnosis cardiac amyloidosis, light chain amyloidosis

## Abstract

Light chain (AL) amyloidosis is caused by a plasma cell clone that produces a dysfunctional, monoclonal light chain. This light chain misfolds and aggregates, resulting in catastrophic organ damage and eventual death. Due to the multiple organs affected by the disease, symptoms can be vague, such as lethargy, fatigue, weight loss, and peripheral edema. This case presentation follows a 66-year-old female patient who presented to our emergency department complaining of progressive shortness of breath (SOB). The patient denied any other associated symptoms and denied any past significant medical history. The lack of other associated symptoms or past diagnoses was a part of this atypical presentation, as the patient frequently complained of a multitude of symptoms affecting more than one organ system, as the pathogenesis of the disease is systemic in nature. Additionally, individuals with undiagnosed amyloidosis have often been diagnosed with other disease processes based on their symptomatology, due to their sometimes nonspecific nature. Upon further evaluation, bilateral pleural effusions were noted on imaging, which was treated with a pigtail pleural catheter. Atrial fibrillation was noted on ECG and was treated with both rate- and rhythm-controlled amiodarone. AL amyloidosis was suspected on transthoracic cardiac ultrasound, which was described as a "grainy" appearance of the myocardium, along with heart failure and left ventricular hypertrophy, supported by serum protein electrophoresis (SPEP) and urine protein electrophoresis (UPEP) results. This was confirmed by abdominal fat pad and bone marrow biopsies. While the use of cardiac ultrasound in the preliminary workup of amyloidosis is supported by evidence-based practice, there is a need for confirmatory diagnostic testing. This led to the decision to utilize fat pad biopsy as the primary definitive diagnostic tool, as it was readily available within the hospital system and was technically easier than many of the alternatives. However, this is neither supported nor refuted as the gold standard by the available literature. The patient was transferred to a higher level of care at a center specifically focused on the treatment of amyloidosis to receive the current gold standard of treatment, which was unavailable at our facility due to a lack of these specialized medications, as well as a provider comfortable with the use of these treatments.

## Introduction

Amyloid light chain (AL) amyloidosis represents approximately 70% of all cases of systemic amyloidosis. Amyloidosis, in general, has an incidence of approximately one in 100,000 individuals worldwide, with approximately 80% of those new cases being AL in nature. The pathogenesis of this condition is due to a plasma cell clone that produces a dysfunctional, monoclonal light chain protein. This light chain misfolds and aggregates, causing catastrophic organ damage and eventual death. The only spared organ system is the central nervous system. This light chain found in AL amyloidosis is distinct from that of the one found in multiple myeloma in terms of both size (the one found in AL amyloidosis is typically smaller) and genetic drivers. The genetic driver found in approximately half of the cases is a t (1:14) that involves cyclin D1 and IgH. This translocation is commonly seen in oncologic and oncologic-like processes, as it leads to uncontrolled cell division without checkpoint inhibition. Individuals who are found to have this abnormality in the setting of amyloidosis have lower hematological response rates, higher relapse rates, and shorter overall survival when treated with certain pharmacological regimens [1]. While this translocation can be seen in multiple myeloma, it is far less common than amyloidosis. It is believed that this is because plasma cells rely on the proteasome to cope with the proteotoxicity exerted by the misfolded, amyloidogenic light chains. However, this also represents an explanation for why those with AL are found to gain therapeutic benefit from treatment with proteasome inhibitors. 

The most common presentation of AL amyloidosis is cardiac involvement, which manifests as heart failure with preserved ejection fraction due to amyloid deposits within the myocardium. This leads to typical echocardiographic findings of left ventricular hypertrophy with associated left atrial enlargement, granular sparkling (sometimes described as a grainy appearance), and longitudinal strain. Low voltages are also noted on the ECG. It is important to note that the degree of cardiac involvement is the main determinant of prognosis and survivability [2]. The disease process has a 10-year survival rate of 20% with treatment, which drops down to about 5% without treatment. As the degree of cardiac involvement in a patient increases, the associated mortality rate rises exponentially. An additional metric that can be considered is N-terminal pro-B-type natriuretic peptide (NT-proBNP), which is a marker of advanced heart failure [3]. This has been found to be an indicator of the prognosis and severity of amyloidosis, particularly in cases with known or suspected cardiac involvement. 

As AL amyloidosis affects multiple organ systems, its renal involvement results in albuminuria, which can then progress to nephrotic syndrome. Along with these more localized manifestations, constitutional symptoms such as lethargy, fatigue, weight loss, and peripheral edema are also commonly noted [4]. Additionally, bilateral carpal tunnel syndrome is commonly seen in the past medical history of a patient with AL amyloidosis. While there is no histological evidence of this, the most widely accepted rationale is that there is small amount of amyloid deposition in ligamentous structures, including but not limited to, the transverse carpal ligament, which then causes a clinical presentation very similar to carpal tunnel syndrome. 

The diagnosis of AL amyloidosis is largely dependent on histological preparation and analysis. Biopsy of any involved tissue can yield the necessary results to establish its diagnosis. That being said, it has been found that “biopsy of the bone marrow combined with abdominal subcutaneous fat aspiration will identify amyloid deposits in 85% of patients with amyloidosis” [5]. Once the diagnosis has been established, it is important to differentiate whether it is a local or systemic deposition. Local sites of deposition involve the skin, bowel and urinary tract, including the kidney, ureter, bladder, and urethra. The symptoms associated with this deposition can be treated symptomatically without the need for systemic therapies. However, in most cases of localized depositions, there already is or will be accompanying systemic deposition. Thus, in the majority of amyloidosis cases, systemic therapy is required for disease remission. 

As previously mentioned, the main prognostic factor is the degree of cardiac involvement. In addition, the free light chain (LC) levels at the time of diagnosis, the number of organs implicated, as well as the serum uric acid level are directly proportional to worse clinical outcomes [6]. 

## Case presentation

Chief complaint 

A 66-year-old female patient (never smoker) presented to our emergency department, complaining of increasing shortness of breath (SOB). 

History of presenting illness 

The patient’s SOB had begun approximately one month prior to presenting and was accompanied by a non-productive cough. Five days before her presentation, she had been assessed by her primary care provider (PCP) due to progressive dyspnea on exertion. She was now unable to complete activities of daily living, although she had maintained complete independence a month prior to symptom onset. A chest X-ray (CXR) was taken at that time and showed bilateral pleural effusions. Upon presenting to the emergency department, the patient stated that she had a fever of 103 degrees Fahrenheit. She endorsed SOB at rest, and this necessitated 2L oxygen via nasal cannula to maintain oxygen saturation of 92-96%. 

Past medical history 

The patient only endorsed hypercholesterolemia and chronic back pain, which is believed to be non-contributory to the presenting case. Five years prior to presentation, the patient had bilateral carpal tunnel syndrome that improved with wrist-splinting.

Medication history 

Her pre-admission medication regimen included amoxicillin, budesonide/formoterol, and prednisone, which the patient had been prescribed five days before by her PCP, when she was found to have bilateral pleural effusions. Additionally, she was on tramadol 50 mg PO every six hours (Q6H) for back pain, alprazolam 0.5 mg PO daily pro re nata (PRN) or as needed for anxiety, and atorvastatin 40 mg PO daily.

Physical examination at time of admission

Pulmonary examination elicited bilateral crackles (right greater than the left), best heard at the lower lung bases and decreased breath sounds on the left lower lung bases on auscultation. Additionally, bilateral dullness to percussion was appreciated. The patient was noted to be in normal sinus rhythm at the time of admission. Bilateral non-pitting edema was present at the level of the mid-shin. 

Laboratory examinations 

Routine laboratory findings (Table 1) were significant for thrombocytopenia, leukocytosis, and hypocalcemia.

**Table 1:** Results of the laboratory investigations

Parameter	Measured value	Reference value
Complete blood count		
White Blood Cell Count (x10^3^μL)	11.6	4-10
Platelet Count (x10^3^μL)	121	150-450
Basic metabolic panel		
Calcium (mg/dL)	7.5	8.3-10.6
Serum Protein Electrophoresis (SPEP)		
Alpha-2-globulin (g/dL)	1.2	0.4-1.2
Beta-globulin (g/dL)	0.6	0.7-1.3
Beta-2-microglobulin (g/dL)	4.7	1.2-2.4
Gamma-globulin (g/dL)	0.2	0.4-1.8
Free Kappa Light Chain (LC) (mg/L)	7	3.3-19.4
Free Lambda Light Chain (LC) (mg/L)	2026	5.7-26.3
Kappa/Lambda ratio	0	0.26-1.65
M-Spike	Not observed	
Urine Protein Electrophoresis (UPEP)		
Free Kappa Light Chain (LC) (mg/L)	136.3	1.17-86.46
Free Lambda Light Chain (LC) (mg/L)	4558.56	0.27-15.21
Kappa/Lambda ratio	0.03	1.83-14.26
M-Spike	Not observed	

Initially, the patient was found to have an elevated high-sensitivity cardiac troponin (hs-cTn) of 326 ng/L (reference range: <14 ng/L) within the ED. Subsequent measurements at 3, 24, and 48 hours after presentation yielded similarly elevated levels of 283 ng/L, 249 ng/L, and 266 ng/L, respectively. However, there was no ischemic changes seen on electrocardiography. 

Serum protein electrophoresis (SPEP) showed hypoalbuminemia, elevated alpha-2-globulins, low beta-globulin, elevated beta-2-microglobulin, and low gamma-globulin. Additionally, there was no M-spike observed. This is consistent with non-selective protein loss. Serum free Kappa LC was normal; however, Lambda LC was extremely elevated. This gave a Kappa/Lambda ratio of 0 (Table 1). This contrasted with urine protein electrophoresis (UPEP) which showed an elevated Kappa LC and an extremely high Lambda LC, resulting in a low Kappa/Lambda ratio of 0.03. There was also no M-spike found on UPEP either (Table 1). 

Imaging examinations 

**Figure 1:**
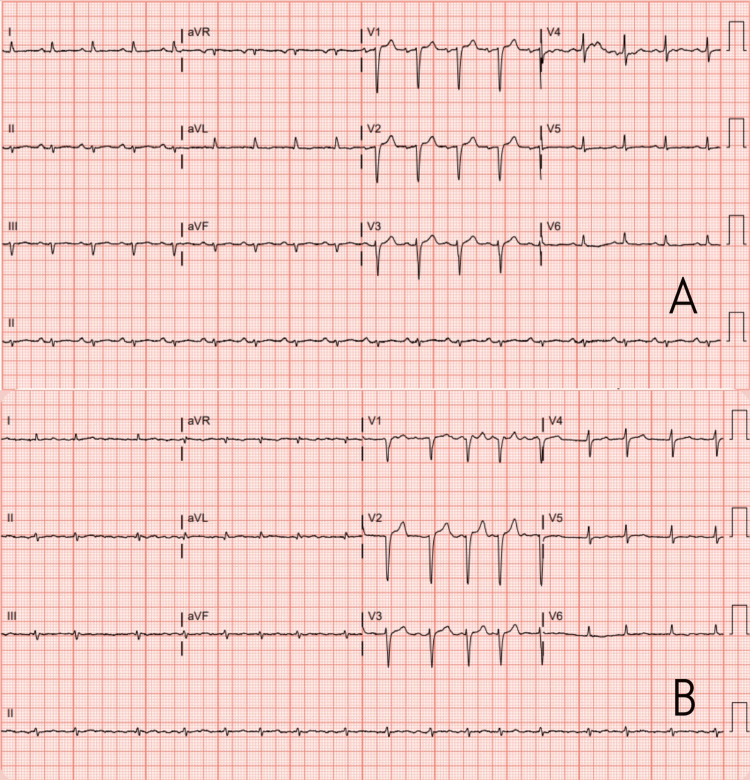
Electrocardiogram at presentation within the ED (A) and then upon admission to the progressive care unit (PCU; B) The findings of low voltage QRS along with non-specific T and R wave abnormalities are both classic, though non-specific findings for amyloidosis. The progression to A-fib with rapid ventricular response (RVR) along with worsening symptomatology further supports cardiac involvement in this patient, even in the absence of confirmatory cardiac muscle biopsy.

The initial ECG showed sinus tachycardia with a left axis deviation, low voltage QRS, and a nonspecific T wave abnormality (Figure 1A).

However, a subsequent ECG showed underlying atrial fibrillation with slight rapid ventricular response (RVR) and poor precordial R wave progression (Figure 1B). 

**Figure 2:**
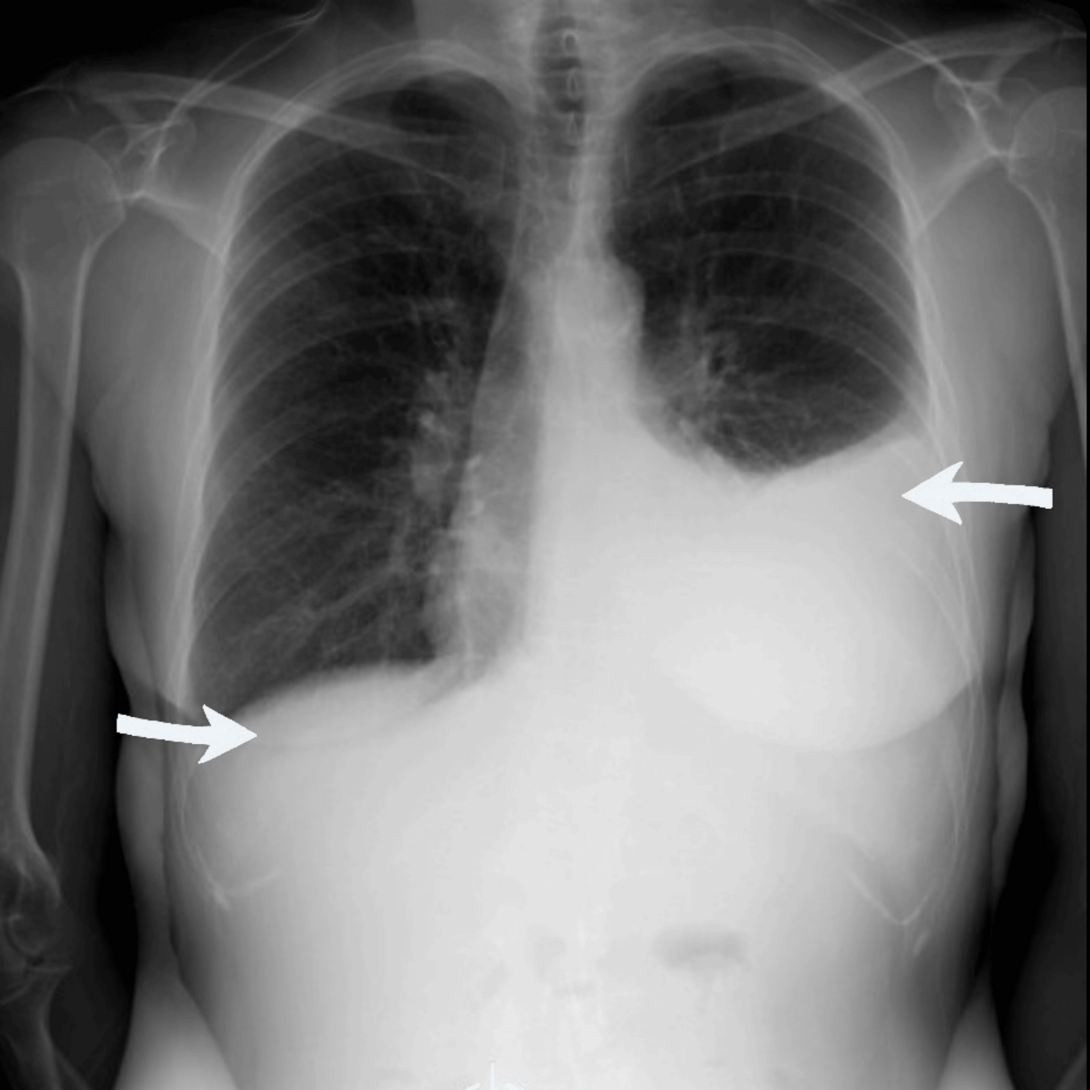
Chest X-ray at time of presentation demonstrating a large left and a smaller right pleural effusion, as denoted by the arrows While this finding did not necessarily raise clinical suspicion for amyloidosis, it did provide a rationale for the presenting shortness of breath and also led to the further workup that eventually led to the confirmatory diagnosis.

An initial chest radiograph showed a large left pleural effusion, as well as a small right pleural effusion (Figure 2).

A subsequent CT angiogram also showed bilateral dependent atelectasis/infiltrate (left greater than right). 

**Figure 3:**
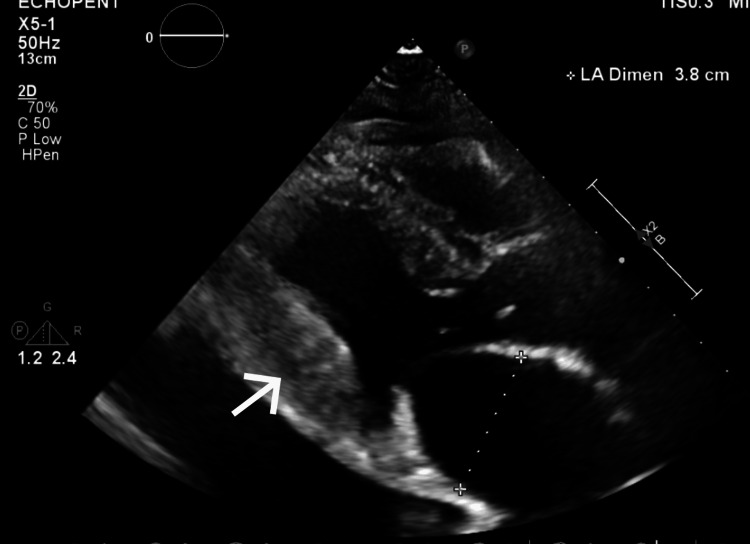
Transthoracic echocardiogram demonstrating a grainy appearance to the myocardium, as denoted by the white arrow This interpretation by the cardiologist first raised the clinical suspicion for amyloidosis. This finding, along with the increased left atrial enlargement (demarcated by the dotted line) and the corresponding left ventricular hypertrophy (also denoted by the arrow), showed a pattern typically seen in amyloidosis.

The echocardiogram showed moderate left ventricular hypertrophy with a low normal left ventricular systolic function (ejection fraction: 51%), as well as grade 2 diastolic dysfunction. Additionally, a reduced global longitudinal strain of 15% was noted. It is also important to note that the texture of the myocardium appeared grainy (Figure 3), which could be indicative of an infiltrative process. 

Medical management 

A left pigtail pleural catheter was placed for the treatment of the larger left pleural effusion. The cumulative drainage of the chest tube was 3615 mL of fluid that was sent for pleural fluid analysis and culture. The cytological analysis showed reactive mesothelial cells in a background of white blood cells and red blood cells. The pleural fluid culture showed Staphylococcus epidermidis, which was believed to be a contaminant in the absence of any signs of infectious disease. 

The A-fib with RVR was treated with amiodarone, which provided both rate and rhythm control. The use of anticoagulation was discussed, however, it was found to be contraindicated due to the patient’s thrombocytopenia, and the presence of a chest tube. The patient was on pharmacologic deep vein thrombosis (DVT) prophylaxis with enoxaparin.

Significant proteinuria was found on urinalysis and treated with the administration of albumin, which partially resolved this issue. Additionally, the non-pitting edema in the calves was treated with furosemide.

**Figure 4:**
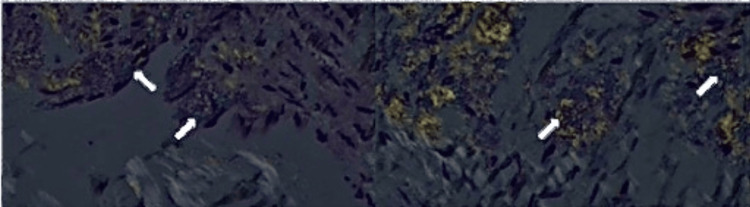
Congo Red staining of the patient's abdominal fat pad biopsy The presence of the areas of apple green birefringence under polarized light (denoted by the arrows) is significant, as the Congo Red staining reagent preferentially binds to misfolded protein chains, characteristically seen in amyloidosis. Therefore, this finding helped confirm the diagnosis, which was previously only suspected based on serum markers and imaging.

A fine needle aspiration (FNA) of the abdominal fat pad was performed under the suspicion of amyloidosis, and subsequently a bone marrow biopsy was done. FNA showed the presence of amyloid with no cytomorphologic findings, suggestive of a plasma cell neoplasm (Figure 4).

This was correlated with the bone marrow biopsy which showed unremarkable trilineage hematopoiesis with plasma cells comprising approximately 5% of total cells counted. Both results were followed-up on an outpatient basis with the Amyloidosis Center at the Boston Medical Center. It was recommended that the patient receive daratumumab-cyclophosphamide, bortezomib, and dexamethasone (CyBorD) for six cycles and continue on daratumumab for two years after that.

## Discussion

In this case, the first clinical suspicion of AL amyloidosis was on echocardiogram, which portrayed that the myocardium had a “grainy” quality. While few published works describe the "grainy" appearance explicitly, previous studies have demonstrated that in almost all cases of amyloidosis, there are findings of left ventricular hypertrophy and left atrial enlargement, as well as a borderline reduced ejection fraction on echocardiogram. These findings were also observed in this case report [7]. The literature supports echocardiography as a preliminary diagnostic tool to increase clinical suspicion and serve as a rationale for further testing. However, it has not proven to be sufficient to definitively diagnose AL amyloidosis, much less cardiac amyloidosis [8]. This was precisely the role that the echocardiogram played within the presented case, with cardiac amyloidosis never being diagnosed definitively. 

Another consideration in the case was the first definitive diagnostic testing. The patient was given the choice of fat pad biopsy and bone marrow biopsy, and counselled with respect to the risks, benefits, as well as rates of false positive or false negative in both modalities. The patient elected to undergo a fat pad biopsy first, and it was later determined that a bone marrow biopsy should be performed. The literature does not endorse one diagnostic test over another, as both modalities show similar sensitivity and specificity for the diagnosis of systemic amyloidosis, with metrics of around 85% and 91%, respectively [9]. Many studies support fat pad biopsy as the primary test of choice, as it provides good sensitivity and specificity, while being one of the most accessible tools for work-up [10]. Interestingly, in our case, the bone marrow biopsy following the fat pad biopsy was found to be negative. There was no amyloid deposition found within the sample of bone marrow tested. As with all pathologic biopsy samples, it is susceptible to a variety of sources of error, including the quality of the sampling technique, sample preparation, as well as the staining process. However, the presence of one positive biopsy result is sufficient to make the diagnosis of amyloidosis, even in the setting of discordant results from other tests, with the presence of LC further confirming the diagnosis as AL amyloidosis [11].

An alternative confirmatory test that has largely been considered the gold standard for the presence of cardiac amyloid deposition is cardiac MRI. This is in large part due to its nearly 100% specificity and non-invasive nature, compared to the aforementioned confirmatory tests. However, despite this, cardiac MRI is not routinely done, as one study showed that only approximately one-third of all patients suspected of having cardiac amyloidosis underwent this test [7]. This is concordant with the decision in this case report. The limited availability of MRI capabilities, both with respect to technologists as well as the MRI machine itself, significantly affected the diagnostic pathway and contributed to the decision to move forward with the fat pad biopsy.

When considering the prognostic factors associated with clinical outcomes, one accepted method of stratification is the Mayo Criteria for Light Chain (AL) Amyloidosis [12]. It features the utilization of the difference between involved and uninvolved light chain (FLC-diff), cardiac biomarkers troponin-T (cTnT) and NT-proBNP, and is scored between 0-3, with an increase in stage with every increase in points [13]. Based on this criteria, the patient discussed here was a stage III, and was given a score of 2 for significant elevations in the FLC-diff and cTnT. This further supports both the diagnosis and severity of amyloidosis seen in this case report. 

It was collectively decided that the patient should be transferred to a specialized level of care for further management and treatment. This decision was made in large part due to the lack of the gold standard confirmatory test, the lack of a specialist comfortable with the additional testing required to pinpoint the exact form of amyloidosis, as well as the treatment regimen described above. An important point to be noted was that the hospital formulary also did not have the gold standard of treatment. A daratumumab-based regimen has consistently been considered the current first line of therapy for AL amyloidosis with or without cardiac involvement. Daratumumab targets one of the specific proteins expressed on the surface of the pathologic plasma cells seen in amyloidosis. Complete hematological response, as measured by decrease in the concentration of amyloid proteins in circulation to below a measurable level, has been to be significantly improved by the use or addition of daratumumab. Additionally, there was a significant reduction in the rate of relapse [14]. This supports the decision to transfer the patient based on availability of cutting-edge pharmacological treatments.

## Conclusions

This case presentation illustrates the sometimes elusive nature of amyloidosis as a diagnosis. The lack of symptoms corresponding to multiple organ systems, along with the lack of relevant past medical history that would align with the typical presentation and medical background of a patient suffering from undiagnosed amyloidosis, was one of the major contributing factors to the atypical nature of this case. An early consideration of amyloidosis within the differential diagnosis could improve the prognosis and quality of life of the patient, given its progressive nature. This could lead to the earlier initiation of disease-modifying therapies, such as the daratumumab-based regimen, which has been previously shown to delay organ deterioration and prolong disease-free living. Additionally, it is important to understand that the symptomatology of the patient may not always match the involved organ, as seen in this case study. There was pretty significant cardiac involvement, as noted by the echocardiogram findings and the serum cardiac markers. Through this case study, we hope that there is continued improvement in diagnostic suspicion for amyloidosis, even in atypical clinical presentations. This is especially true for individuals suffering from heart failure without a plausible underlying etiology or sudden or unexplained pleural effusions, as seen in this case report. 
